# Lipoprotein (a) is related to In-Stent neoatherosclerosis incidence rate and plaque vulnerability: Optical Coherence Tomography Study

**DOI:** 10.1007/s10554-022-02736-3

**Published:** 2022-10-31

**Authors:** Xiaohang Yuan, Yan Han, Xin Hu, Mengting Jiang, Huanhuan Feng, Yan Fang, Miao Liu, Yundai Chen, Lei Gao

**Affiliations:** 1 Senior Department of Cardiology, the Sixth Medical Centre of Chinese PLA General Hospital, No. 6 Fucheng Road, Haidian District, 100853 Beijing, China; 2 Department of Emergency, the First Medical Centre, Chinese PLA General Hospital, No. 28 Fuxing Road, Haidian District, 100853 Beijing, China; 3 Department of Statistics and Epidemiology, Graduate School, PLA General Hospital, No. 28 Fuxing Road, Haidian District, 100853 Chinese, Beijing, China

**Keywords:** Optical coherence tomography, In-stent restenosis, In-stent neoatherosclerosis, Thin-cap fibroatheroma

## Abstract

**Background:**

In-stent neoatherosclerosis (ISNA) is an important reason for stent failure. High lipoprotein (a) [Lp (a)] level is an independent predictor of in-stent restenosis (ISR). To date, the relationship between the level of serum Lp (a) and the incidence rate and vulnerability of ISNA has never been verified.

**Methods:**

A total of 119 patients with 125 drug-eluting stent ISR lesions who underwent percutaneous coronary intervention guided by optical coherence tomography were enrolled in this study. According to their Lp (a) level, the patients were divided into two groups [high Lp (a) group ≥ 30 mg/dL, n = 47; or low Lp (a) group < 30 mg/dL, n = 72]. The clinical baseline, angiographic characteristics, and optical coherence tomography data of both groups were recorded and analyzed.

**Results:**

No significant differences in clinical and angiographic characteristics were found between the two groups (*P* > 0.05). The incidence rate of ISNA in the high Lp (a) group was significantly higher than that in the low Lp (a) group (94.0% [n = 47] vs. 52.0% [n = 39], *P* < 0.001). The incidence rate of thin-cap fibroatheroma in ISR lesions was significantly higher in the high Lp (a) group than in the low Lp (a) group (42% [n = 21] vs. 5.3% [n = 4], *P* < 0.001).

**Conclusion:**

A high Lp (a) level is associated with the high incidence rate and plaque vulnerability of ISNA.

**Supplementary Information:**

The online version contains supplementary material available at 10.1007/s10554-022-02736-3.

## Introduction

In-stent neoatherosclerosis (ISNA) is one of the types of in-stent restenosis (ISR) and is an important cause of late stent thrombosis and late ISR. Neoatherosclerosis (NA) is characterized by the accumulation of lipid foamy macrophage within neointima, with or without necrotic core, and the presence of calcium [1] within the culprit stent. NA is also an important indicator of plaque vulnerability. The lipoprotein family plays an important role in many risk factors of NA. Modifying the lipid profile can suppress atherosclerotic progression in de-novo atherosclerosis and NA [2].

Lipoprotein (a) [Lp (a)] is a serum lipoprotein whose structure is composed of apolipoprotein (a) [Apo (a)], apolipoprotein (b) [Apo (b)], and low-density lipoprotein (LDL)-like segment [3]. Lp (a) can promote the formation and development of atherosclerosis and thrombosis and increase the risk of cardiovascular diseases, especially acute coronary syndrome [4]. This function may be related to its participation in lipid metabolism, coagulation, and fibrinolysis system and its stimulation of smooth muscle cell proliferation [5, 6].

The correlation between Lp (a) and adverse clinical outcomes, such as ISR after percutaneous coronary intervention (PCI), remains controversial. Most previous studies were conducted on patients with PCI receiving bare metal stent (BMS) [7]. Recent reports have shown that high Lp (a) levels are significantly associated with the plaque vulnerability of de-novo atherosclerosis, and the same is true in patients with high levels of LDL cholesterol [8]. However, the relationship between the level of serum Lp (a) and the incidence rate and vulnerability of ISNA in ISR patients with drug-eluting stent (DES) implantation has never been verified. Thus, we analyzed the relationship between serum Lp (a) level and ISNA incidence rate and vulnerability in DES-ISR lesions using intracoronary optical coherence tomography (OCT).

## Methods

### Study Population

**Fig. 1:**
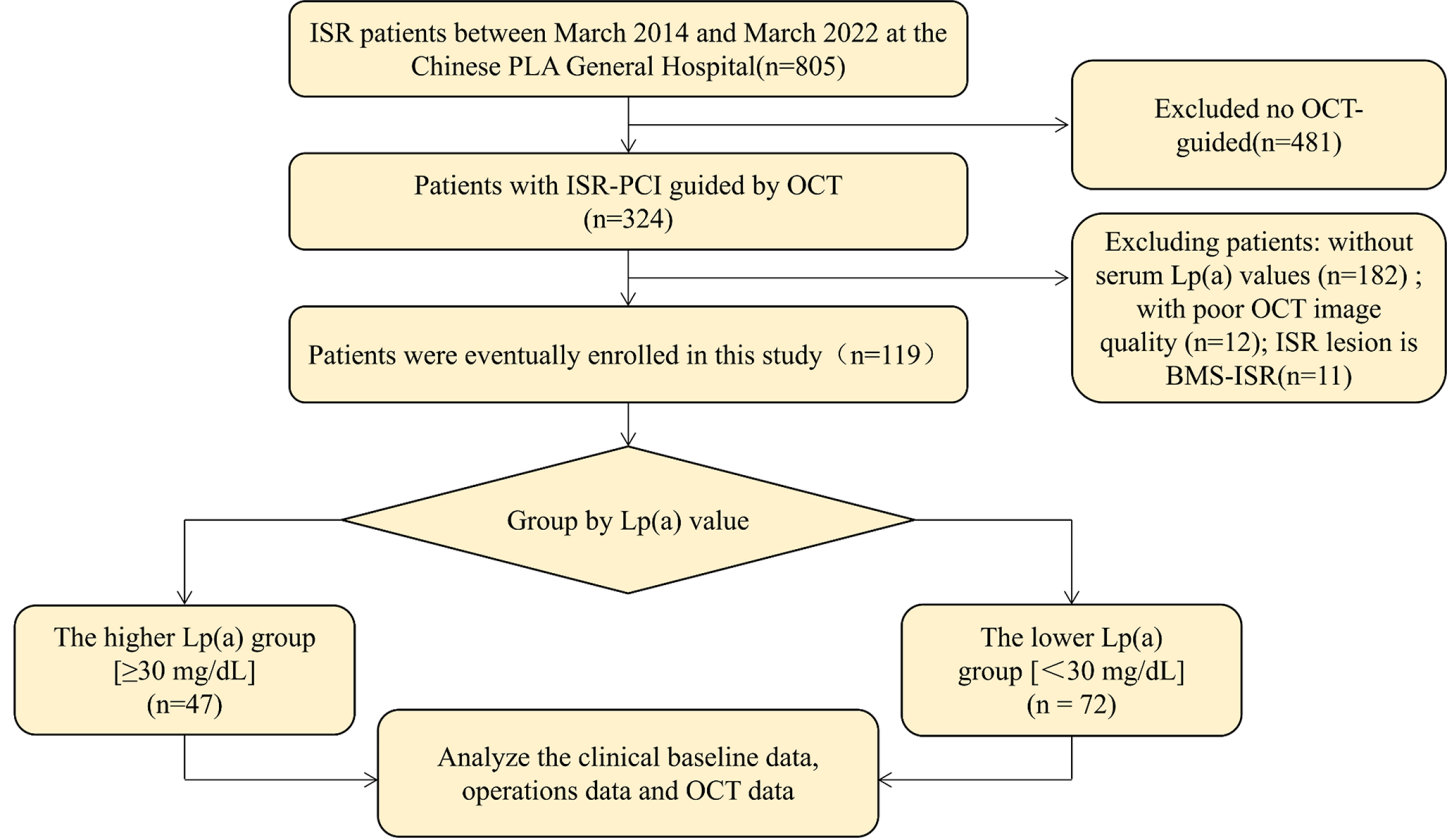
**Study flow** According to their Lp (a) level, the patients were divided into two groups (high Lp (a) group ≥ 30 mg/dL, n = 47; or low Lp (a) group < 30 mg/dL, n = 72)

This single center, retrospective observational study enrolled patients hospitalized at the Chinese People’s Liberation Army (PLA) General Hospital from March 2014 to March 2022. From a total of 805 ISR patients with DES implantation, we included 324 patients treated with OCT-guided ISR lesions. We then excluded patients with BMS-ISR (n = 11), without serum Lp (a) values (n = 182), and with poor OCT image quality (n = 12). Finally, 119 patients with 125 DES-ISR lesions were included in this study (Fig. 1). We divided the enrolled patients into two groups according to their serum Lp (a) level [high Lp (a) group (≥ 30 mg/dL), n = 47; or low Lp (a) group (< 30 mg/dL), n = 72] [9]. The Human Research Committee of the Chinese PLA General Hospital approved this study protocol, and all patients provided written informed consent prior to PCI.

### Clinical baseline and angiographic characteristic data of patients

The baseline data were collected from the medical record system and analyzed by the first author of this paper. The target lesion characteristics of the patients undergoing PCI angiography in the Imaging Core Laboratory of the Chinese PLA General Hospital were collected and analyzed by two independent technicians. Quantitative coronary angiography (QCA) analysis was performed in the core laboratory of the Chinese PLA General Hospital, where QAngio XA software (Medis Medical Imaging Systems, Leiden, the Netherlands) was used by a blinded trained technician to analyze the angiographic images of all patients. Mehran classification was used for ISR classification [10]: type I, focal type (≤ 10 mm), which was subdivided into connected type, marginal type, single focal type, and multi-focal type; type II, diffuse in-stent (> 10 mm, confined to the stenotic stent); type III, proliferative type (> 10 mm, over the edge of the stent); and type IV, complete occlusion.

### OCT Image Acquisition and Assessment

ISR lesions were imaged using OCT catheters (Dragonfly™ Duo; St. Jude Medical, St. Paul, MN, USA) and frequency-domain OCT (ILUMIEN™ OPTIS™ OCT imaging systems; St. Jude Medical), and the OCT catheter was introduced into distal ISR lesions with a pullback rate of 18 mm/s and rotation rate of 100 frames/s [11]. During pullback, the contrast medium was injected through the guiding catheter at a rate of 3–4 mL/s. Qualitative analyses of OCT images were performed by two experienced physicians (Yan Han and Mengting Jiang) who were blinded to clinical and angiographic lesion characteristics. When the two observers had different opinions, they reached a final decision after discussion. To evaluate interobserver differences in OCT diagnosis of ISNA and thin-cap fibroatheroma (TCFA), all OCT images were analyzed using offline proprietary software (St. Jude Medical) by two independent cardiologists (Yan Han and Mengting Jiang) who had no knowledge of the baseline clinical and angiographic characteristics. In addition, one of the two observers (Yan Han) re-evaluated all OCT images 4 months after the initial evaluation to assess intra-observer variability in OCT results in the diagnosis of patients with ISNA and TCFA.

### Definitions

For quantitative and qualitative analyses, OCT cross-sectional images between proximal and distal reference lumen areas were analyzed at 1-mm intervals. For qualitative indicators, the presence of target features in any section can be considered as the existence of this feature. For quantitative indicators, minimum stent area (MSA) in ISR lesion area was analyzed. The main indicators analyzed were the plaque structural characteristics and multiple luminal area characteristics of ISR. For quantitative analysis, the percentage of maximum neointimal hyperplasia (NIH) was calculated as ([stent–lumen area]/stent area) × 100%. Proximal and distal reference lumen areas were the slices with the largest lumen area within 5 mm proximal and distal to the stent edges, respectively. Underexpansion was defined as minimum MSA/the mean of the distal and proximal area < 80% [12].

For qualitative analysis, the accumulation of lipid foamy macrophage within neointima (with or without necrotic core) in the presence of calcium within the culprit stent was defined as NA [13]. The three types of restenotic tissue structure are as follows: (1) homogeneous, which has uniform optical properties and does not show focal variations in backscattering patterns; (2) heterogeneous, which has focally changing optical properties and shows various backscattering patterns; and (3) layered type, which has different optical properties and shows concentric layers consisting of an abluminal high scattering layer and an abluminal low scattering layer. The high type of restenotic tissue backscattering shows high backscattering for the majority of tissues and appears bright. The low type of restenotic tissue backscattering shows low backscattering for most tissues and appears dark or black.

**Fig. 2:**
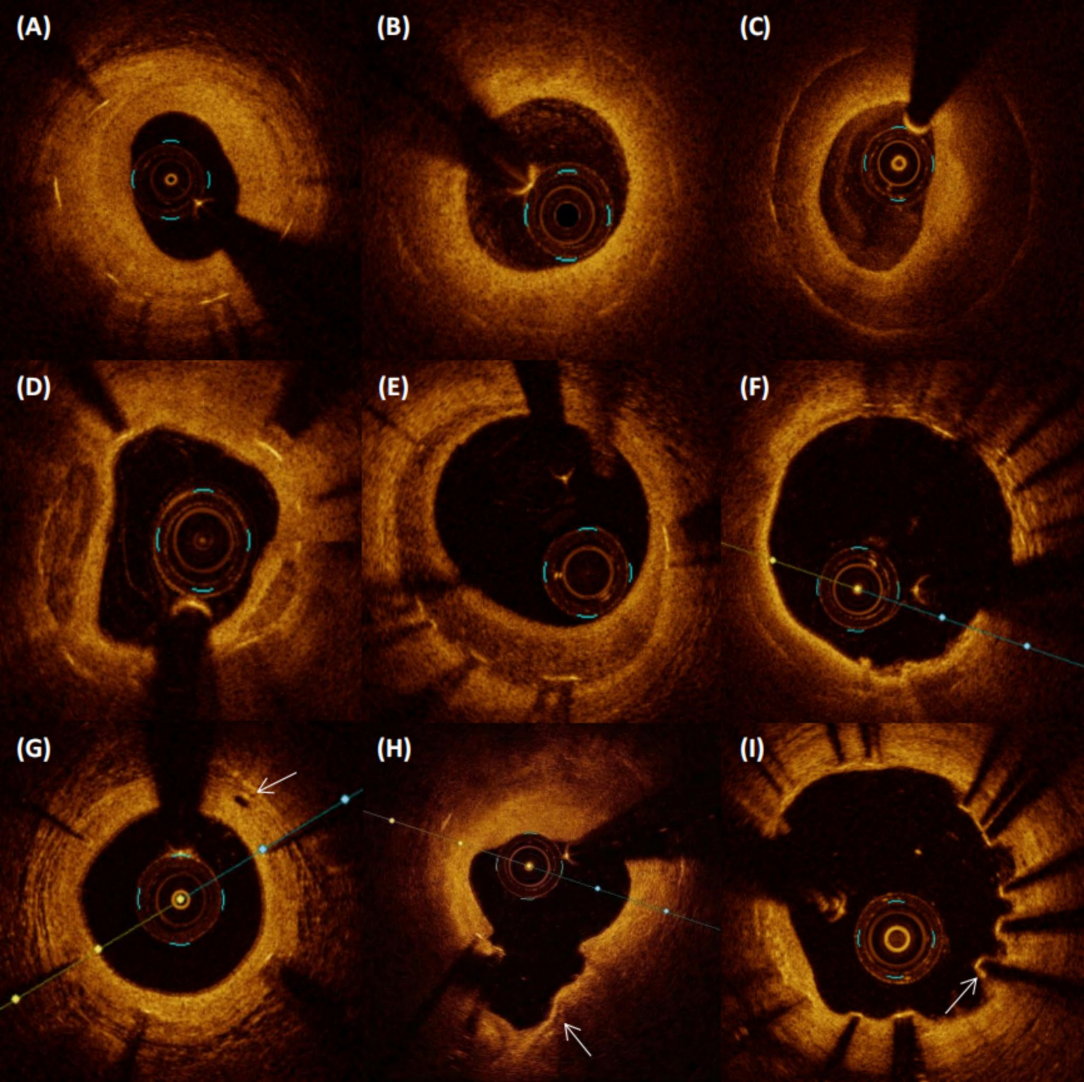
**Representative optical coherence tomography images of various vascular morphologies** Various classical images of OCT. (A) Homogenous hyperplasia, high-intensity. (B) Heterogeneous, low-intensity. (C) Layered, low-intensity. (D) Neointimal calcium. (E) Layered, high-intensity. (F) Lipidic neointimal hyperplasia, thin-cap fibroatheroma. (G) Microvessels (arrows). (H) Neointimal rupture (arrow). (I) Malapposition (arrow)

In the OCT images, lipid plaque was defined as a signal-poor diffuse region, and the TCFA in lipid plaques was ≤ 65 μm [14]. Neointimal calcium was classified as a poorly signalized and well-delineated region. Plaque rupture was described as the rupture of the fibrous cap that connects the lumen to the underlying lipid NIH. A malapposed strut was defined as a strut whose measured distance between its surface and the adjacent vessel surface is greater than the sum of the thickness of the strut plus polymer [15]. Various classical OCT images are shown in Fig. 2.

### Statistical analysis

For measurement data with normal distribution, mean *± SD* was used for description, and a comparison between the two groups was performed by *t* test. For data with skewed distribution, the median was used for description, and the rank-sum test was used for comparison between the two groups. Count data were expressed as rates (%), and *the χ*^*2*^ test was used to compare the data between the two groups. Univariate and multivariate logistic regression analyses were performed to determine the independent factors for the presence of OCT-ISNA that suggest an association with the presence of ISNA [13]. Statistical significance was defined as *P* < 0.05. Inter- and intra-observer variabilities in diagnostic tissue morphology were assessed by Cohen’s coefficient Kappa. All statistical analyses were performed using SPSS software version 22.0 (IBM Corp, Armonk, NY, USA). OCT-ISNA and OCT-TCFA were set as target events. ROC curves were constructed using Lp (a) values, and the area under the curve (AUC), sensitivity, and specificity were calculated. The best cut-off value of Lp (a) was determined by maximizing the sum of sensitivity and specificity of the ROC curve.

## Results

### Clinical characteristics

A total of 119 patients with ISR and 125 ISR target lesions were included in this study. These patients comprised 96 men and 23 women with a mean age of 63.9 ± 9 years. No significant differences in baseline clinical and angiographic characteristics were observed among these patients (Tables 1 and 2).

**Table 1:** Baseline characteristics

	Overall population (n = 119)	Lp (a)		*P* value
		**Lp (a) < 30 mg/dL** **(n = 72)**	**Lp (a) ≥ 30 mg/dL** **(n = 47)**	
Age, years	63.9 ± 9	64.1 ± 9	63.4 ± 10	0.704
Male	96 (80.7)	58 (80.6)	38 (80.9)	0.968
Current smoker	47 (39.5)	33 (45.8)	14 (29.8)	0.080
Hypertension	73 (61.3)	48 (66.7)	25 (53.2)	0.140
Diabetes mellitus	60 (50.4)	39 (54.2)	21 (44.7)	0.312
Hyperlipidemia	40 (33.6)	25 (34.7)	15 (31.9)	0.751
Chronic renal insufficiency	4 (3.4)	1 (1.4)	3 (6.4)	0.338
Chronic cardiac insufficiency	14 (11.8)	11 (15.3)	3 (6.4)	0.141
Prior coronary artery bypass grafting	0 (0)	0 (0)	0 (0)	–
Medication at the time of in-stent restenosis				
Aspirin	113 (95.0)	66 (91.7)	47 (100)	0.109
Clopidogrel	84 (70.6)	50 (69.4)	34 (72.3)	0.735
Ticagrelor	33 (27.7)	15 (20.8)	18 (38.3)	0.037
Statin	104 (87.4)	62 (86.1)	42 (89.4)	0.601
Fasting blood glucose, mmol/L	5.8 (5.0, 8.5)	5.8 (4.9, 8.6)	5.7 (5.0, 7.2)	0.642
LDL cholesterol, mmol/L	1.9 (1.5, 2.3)	1.8 (1.4, 2.3)	2.0 (1.6, 2.3)	0.185

Values are n (%) or mean and standard deviation or median (1st quartile, 3rd quartile). LDL: low-density lipoprotein

**Table 2:** Angiography characteristics

	Overall population (n = 125)	Lp (a)		*P* value
		**Lp (a) < 30 mg/dL** **(n = 75)**	**Lp (a) ≥ 30 mg/dL** **(n = 50)**	
Time since implantation, years	5.6 ± 5	6.3 ± 5	4.6 ± 4	0.092
Culprit vessel				
Left anterior descending	75 (60.0)	44 (58.7)	31 (62.0)	0.709
Circumflex	22 (17.6)	10 (13.3)	12 (24.0)	0.125
Right	26 (20.8)	19 (25.3)	7 (14.0)	0.126
other	2 (1.6)	2 (2.7)	0 (0)	0.516
Lesion characteristics				
Bifurcation (> 1.5 mm)	34 (27.2)	19 (25.3)	15 (30.0)	0.566
Ostial location	16 (12.8)	7 (9.3)	9 (18.0)	0.155
In-stent restenosis pattern*				0.674
Type I	55 (44.0)	32 (42.7)	23 (46.0)	
Type II	30 (24.0)	18 (24.0)	12 (24.0)	
Type III	33 (26.4)	22 (29.3)	11 (0.22)	
Type IV	7 (5.6)	3 (4.0)	4 (8.0)	
QCA analysis of lesion segment				
Restenosis lesion length, mm	10.2 (7.5, 13.9)	10.3 (7.5, 13.9)	9.7 (7.2, 13.6)	0.325
Reference vessel diameter, mm	2.6 (2.2, 3.0)	2.5 (2.2, 3.0)	2.6 (2.3, 3.1)	0.341
Minimum lumen diameter, mm	1.0 (0.7, 1.4)	1.0 (0.7, 1.3)	1.0 (0.6, 1.4)	0.766
Diameter stenosis,%	60.7 (50.6, 72.3)	60.7 (49.0, 72.1)	60.6 (51.5, 73.2)	0.517

Values are n (%) or mean or median with first and third quartiles. *In-stent restenosis pattern was defined as per Mehran’s classification

### OCT analysis of ISR Lesions

**Fig. 3:**
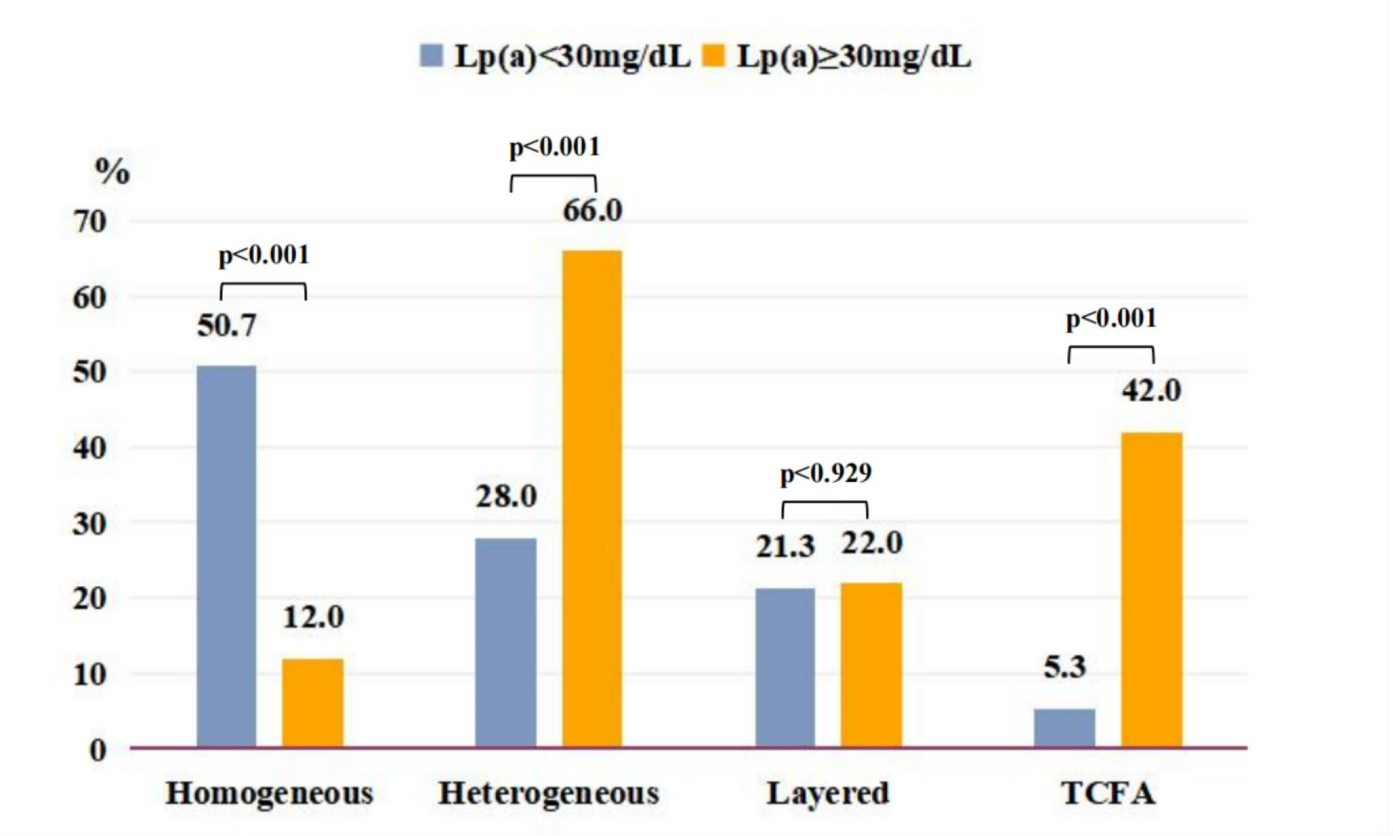
**Pattern of restenotic tissues** More homogeneous neointima(50.7% vs. 12.0%) in the lower Lp (a) group; and more TCFA(42.0% vs. 5.3%) in the higher Lp (a) group (p < 0.05).TCFA: thin-cap fibro-atheroma

A significant difference in the distribution of the pattern of ISR stenotic tissue structure tissue was observed between the two groups: more cases of homogeneous neointima were found in the low Lp (a) group than in the high Lp (a) group (50.7% vs. 12.0%) (*P* < 0.001), and more cases of TCFA were found in the high Lp (a) group than in the low Lp (a) group (42.0% vs. 5.3%) (*P* < 0.001) (Fig. 3). According to the Lp (a) quartile level, the incidence rates of ISNA (Supplementary Fig. 1–A) and TCFA (Supplementary Fig. 1–B) increased with the Lp (a) level. Detailed OCT data based on the Lp (a) quartile level can be found in Supplementary Table 1.

Significantly higher rates of OCT-ISNA (94.0% [n = 47] vs. 52.0% [n = 39], *P* < 0.001) and OCT-TCFA (42.0% [n = 21] vs. 5.3% [n = 4], *P* < 0.001) were found in the high Lp (a) group than in the low Lp (a) group. In addition, a higher prevalence of lipid plaques (92.0% [n = 46] vs. 45.3% [n = 4], *P* < 0.001) and a wider lipid arc [302.8 (219.6, 360.0) vs. 205.8 (136.7, 284.1), *P* = 0.001] were observed in the patients with Lp (a) ≥ 30 mg/dL compared with those with Lp (a) < 30 mg/dL. Microvasculature (44.0% [n = 22] vs. 10.7% [n = 8], *P* < 0.001) and neointimal macrophages (76.0% [n = 38] vs. 33.3% [n = 25], *P* < 0.001) appeared more frequently in the patients with high Lp (a) than in those with Lp (a) < 30 mg/dL (Table 3).

**Table 3:** OCT data

	Overall population (n = 125)	Lp (a)		*P* value
		**Lp (a) < 30 mg/dL** **(n = 75)**	**Lp (a) ≥ 30 mg/dL** **(n = 50)**	
Quantitative assessment				
Distal reference lumen area, mm^2^	5.0 (4.0, 6.4)	5.0 (4.0, 6.3)	5.2 (3.8, 6.6)	0.613
Proximal reference lumen area, mm^2^	6.6 (5.7, 8.5)	6.5 (5.3, 8.2)	6.9 (6.1, 8.9)	0.157
Minimum lumen area, mm^2^	2.0 (1.4, 2.9)	2.2 (1.3, 3.0)	2.0 (1.4, 2.6)	0.809
Minimum lumen diameter, mm^2^	1.6 (1.3, 1.9)	1.6 (1.3, 1.9)	1.6 (1.3, 1.8)	0.826
Minimum stent area, mm^2^	6.5 (5.2, 8.0)	6.3 (4.8, 7.6)	6.7 (5.9, 8.1)	0.108
Maximal NIH, %	68.5 (51.6, 77.1)	64.1 (47.5, 77.2)	69.7 (54.4, 76.5)	0.444
Mean intimal thickness	0.6 (0.4, 0.8)	0.6 (0.4, 0.8)	0.7 (0.5, 0.8)	0.132
Qualitative assessment				
Neoatherosclerosis	86 (68.8)	39 (52.0)	47 (94.0)	< 0.001
Restenotic tissue structure				< 0.001
Homogeneous	44 (35.2)	38 (50.7)	6 (12.0)	
Heterogeneous	53 (42.4)	21 (28.0)	33 (66.0)	
Layered	27 (21.6)	16 (21.3)	11 (22.0)	
Restenotic tissue backscatter				0.001
High	60 (48.0)	45 (60.0)	15 (30.0)	
Low	65 (52.0)	30 (40.0)	35 (70.0)	
Lipid plaque	80 (64.0)	34 (45.3)	46 (92.0)	< 0.001
Maximum lipidic arc, °	270.0 (180.1, 360.0)	205.8 (136.7, 284.1)	302.8 (219.6, 360.0)	0.001
TCFA	25 (20.0)	4 (5.3)	21 (42.0)	< 0.001
Plaque rupture	15 (12.0)	6 (8.0)	9 (18.0)	0.092
Calcific plaque	13 (10.4)	8 (10.7)	5 (10.0)	0.905
Spotty calcification	8 (6.4)	4 (5.3)	4 (8.0)	0.823
Fibrous plaque	39 (31.2)	36 (48.0)	3 (6.0)	< 0.001
Thrombus	20 (16.0)	11 (14.7)	9 (18.0)	0.618
Neointimal macrophages	63 (50.4)	25 (33.3)	38 (76.0)	< 0.001
Microvessels	30 (24.0)	8 (10.7)	22 (44.0)	< 0.001

Values are n (%) or median with first and third quartiles. OCT: optical coherence tomography; NIH: neointimal hyperplasia; TCFA: thin-cap fibroatheroma

### Prediction of ISNA

Data of 125 lesions were used for logistic regression. As shown in Table 4, univariate (OR: 1.049, 95% CI: 1.023–1.075; *P* < 0.001) and multivariate (OR: 1.054, 95% CI: 1.025–1.084; *P* < 0.001) analyses indicated that Lp (a) level was independently associated with the presence of ISNA.

**Table 4:** Univariate and multivariate logistic regression analysis to determine the independent factors affecting the presence of OCT-ISNA

	Univariate analysis	*P* value	Multivariate analysis	*P* value
	**Odds ratio (95% CI)**		**Odds ratio (95% CI)**	
Age > 65 years	0.926 (0.432–1.975)	0.838	0.866 (0.333–2.253)	0.768
Male	1.543 (0.603–3.948)	0.366	1.458 (0.376–5.656)	0.586
Current smoker	0.767 (0.355–1.655)	0.499	0.885 (0.326–2.403)	0.811
Hypertension	0.457 (0.198–1.054)	0.066	0.775 (0.264–2.276)	0.643
Diabetes mellitus	0.738 (0.345–1.579)	0.433	0.900 (0.351–2.304)	0.825
Hyperlipidemia	0.658 (0.300–1.441)	0.295	0.600 (0.225–1.597)	0.306
LDL cholesterol, mg/dL	1.495 (0.819–2.731)	0.191	0.928 (0.445–1.936)	0.843
Absence of statin use at the time of ISR	1.103 (0.360–3.378)	0.864	2.121 (0.495–9.093)	0.311
NIH > 50%	3.906 (1.638–9.315)	0.002	2.358 (0.700–7.940)	0.166
Time since implantation, years	1.048 (0.958–1.147)	0.302	1.118 (0.990–1.263)	0.072
Minimum lumen area, mm^2^	0.762 (0.533–1.091)	0.138	0.735 (0.432–1.252)	0.257
Lp (a), mg/dL	1.049 (1.023–1.075)	< 0.001	1.054 (1.025–1.084)	< 0.001

ISR: in-stent restenosis; NIH: neointimal hyperplasia; LDL: low-density lipoprotein; ISNA: in-stent neoatherosclerosis; OCT: optical coherence tomography

**Supplementary Table 1. OCT analysis of culprit lesions according to Lp (a) quartile**

### ROC Analysis

**Fig. 4:**
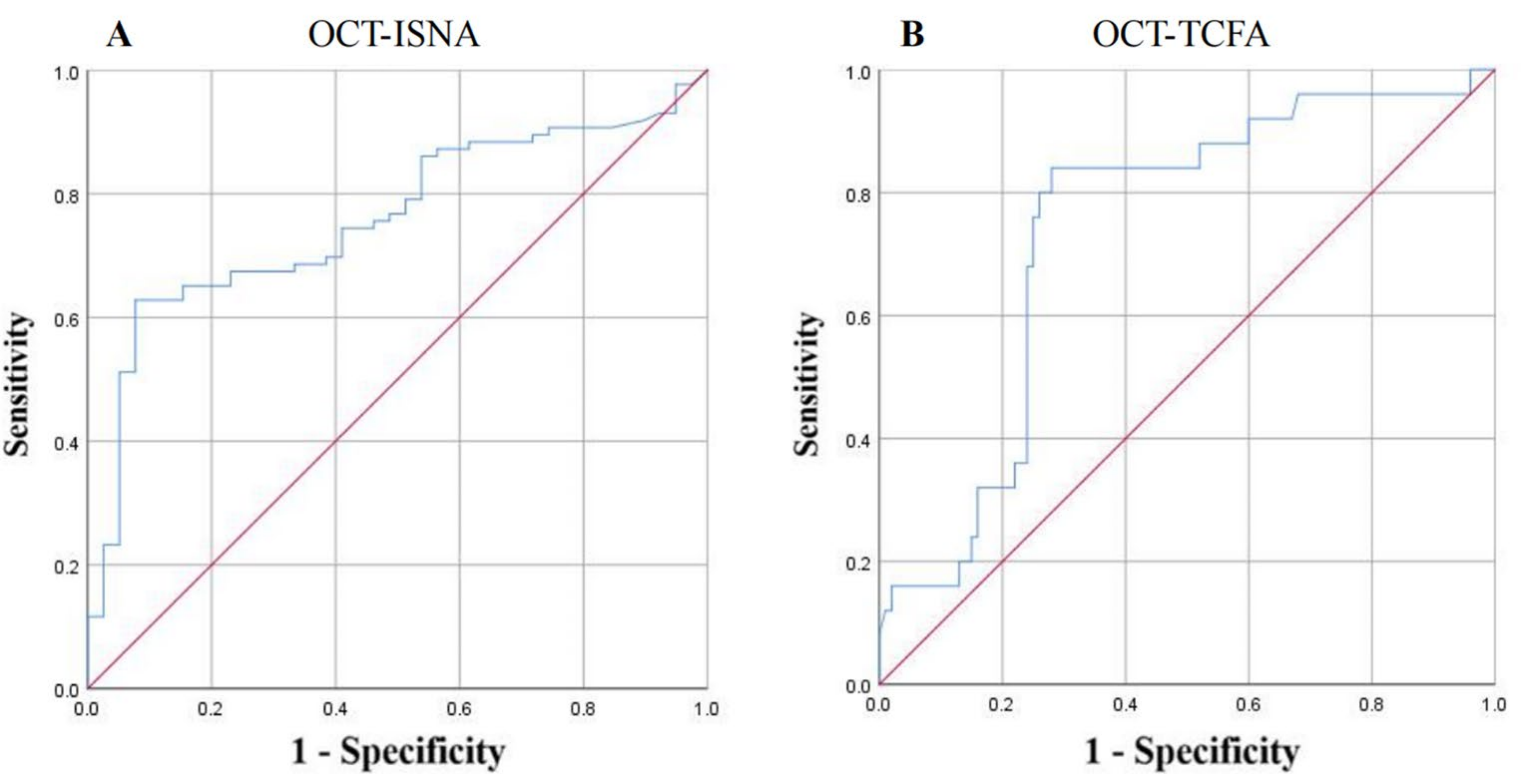
**ROC analysis** ISNA, in-stent neoatherosclerosis; TCFA, thin-cap fibroatheroma

ROC curves were established and analyzed to evaluate the ability of Lp (a) to identify OCT-ISNA (Fig. 4–A) and OCT-TCFA (Fig. 4–B). The AUC was 0.758 (95% CI: 0.672–0.844; *P* < 0.001) for the identification of OCT-ISNA and 0.738 (95% CI: 0.634–0.841; *P* < 0.001) for the identification of OCT-TCFA. According to the ROC analysis results of ISNA and TCFA, the best cut-off of Lp (a) value was 26.6 mg/dL for OCT-ISNA detection (sensitivity 62.8%, specificity 92.3%) and 30.5 mg/dL for OCT-TCFA detection (sensitivity 84.0%, specificity 72.0%).

### Reproducibility of Analysed OCT results

In the qualitative OCT assessment, inter- and intra-observer variabilities (κ value) were 0.89/0.89 for ISNA and 0.91/0.91 for TCFA.

## Discussion

To investigate the relationship between Lp (a) and in-stent NIH characteristics, this study retrospectively analyzed the relationship between serum Lp (a) level and ISR with DES implantation. The main findings on the relationship between Lp (a) and ISNA were as follows: (1) In patients with ISR, the prevalence of ISNA and TCFA was significantly higher in the high Lp (a) group than in the low Lp (a) group; (2) Lp (a) level was independently associated with ISNA plaque vulnerability. Therefore, we infer that a high Lp (a) level is associated with a high ISNA proportion in ISR and increased plaque vulnerability.

This paper focuses on the relationship between Lp (a) and plaque vulnerability under OCT. Many previous studies have demonstrated that plaque vulnerability is associated with adverse clinical outcome events. The CLIMA study examined the predictive value of high-risk plaques on OCT for coronary events within 12 months, The results showed that the minimum lumen area (MLA) < 3.5mm^2^, fiber cap thickness (FCT) < 75 μm, lipid core Angle > 180° and macrophage infiltration under OCT were associated with increased risk of coronary events [16]. The greater the plaque vulnerability, the more clinical adverse events may be caused. Therefore, we tried to find a simple and rapid index to predict plaque vulnerability in the early stage, so as to take active measures to intervene patients.

High Lp (a) values are associated with plaque vulnerability and a high risk of major adverse cardiovascular events in patients with de-novo atherosclerosis [17]. Dahlen et al. studied the high degree of correlation between Lp (a) level and coronary heart disease in Caucasian patients [18]. Kral et al. demonstrated that among healthy African Americans, those with serum Lp (a) > 40 mg/dL were four times more likely to have > 50% coronary stenosis than those with Lp (a) < 40 mg/dL [19]. In addition, Lp (a) is strongly related to the vulnerability of coronary atherosclerosis, leading to the occurrence of cardiovascular events. In their prospective trials, Niccoli et al. also confirmed that a high Lp (a) level was associated with great vulnerability to de-novo atherosclerosis[9]. Hartmann et al. found a positive correlation between Lp (a) values and lipid changes in the plus-media region by intravascular ultrasound [20].

Recently published PACMAN-AMI (Effects of the PCSK9 Antibody Alirocumab on Coronary Atherosclerosis in Patients With Acute Myocardial Infarction) randomized trial has shown that aggressive lipid-lowering leads to degeneration of plaque vulnerability features, including macrophage infiltration [21]. Lp (a) is a member of the lipoprotein family that has received extensive attention recently. However, no studies have discussed the effect of Lp (a) levels on the vulnerability characteristics of ISR plaques, including macrophage infiltration, and no studies have compared the effect of different Lp (a) levels on DES-ISR plaques by OCT. Our results showed that under OCT, the ISR plaque vulnerability of the high Lp (a) group was greater than that of the low Lp (a) group, which was mainly reflected in macrophage infiltration and TCFA. We hypothesized that this might be related to the effect of Lp (a) on ISR plaque progression. The occurrence and progression of ISR and in-situ lesions are similar, and the lipoprotein family plays a key role in both [2]. However, the specific mechanism of Lp (a) in promoting the occurrence and development of de-novo atherosclerotic plaque and ISR plaques has not been fully elucidated. Lp (a) may have the following mechanisms in promoting the progression of de-novo atherosclerotic plaque. First, Lp (a) can penetrate the new intima of the artery and bind to extracellular matrix components, thus promoting macrophage infiltration and smooth muscle cell proliferation [22]. This finding was consistent with the current observation that the high Lp (a) group had higher neointimal macrophage aggregation and a higher proportion of lipid plaques than the low Lp (a) group. In addition, Lp (a) is enriched in plaques at all stages of atherosclerotic lesions and may also play a similar role in ISNA plaques. This result requires further basic experimental pathological analysis for verification. Furthermore, the Apo (a) structure of Lp (a) is crucial for Lp (a) to play its unique role in cardiovascular events [23]. When the intima of the vascular wall is damaged, the pro-inflammatory components under the intima are exposed to blood; Lp (a) moves to the wound site, and its Apo (a), with a similar structure to plasminogen, plays the role of prothrombus at the vascular injury site by inhibiting fibrinolysis [4].

The LDL-like segment of Lp (a) can be phagocytosed by macrophages, which then form foam cells that contribute to the formation and development of atherosclerotic plaques [24]. Lp (a) can also stimulate Lp (a) internalization-mediated macrophage transition to foam cells through very-LDL receptors and Apo (a) receptors [25], and this phenomenon is conducive to the formation and development of atheromatous plaques. Among all lipoproteins, Lp (a) is the largest binding carrier of oxidized phospholipids, which have important pro-inflammatory and pro-atherogenic effects by activating pro-inflammatory signals in endothelial cells, smooth muscle cells, and macrophages and triggering inflammatory responses in arterial walls [26]. We speculated that Lp (a) could also promote the progression of ISNA plaques and increase the vulnerability of ISNA plaques through the above-mentioned inflammatory response.

To date, the pathophysiological mechanism of Lp (a) in promoting ISNA increasing plaque vulnerability has not been proven by relevant basic studies. The current work may provide a new way for the exploration of the pathogenesis and development of ISNA and may contribute to the clinical prevention and treatment of ISNA.

## Limitations

This study has some limitations. First, this research was a retrospective study conducted in a single center with small sample size. When screening the patients included in this paper, more patients did not have OCT examination or Lp (a) detection, so the included patients had a certain selection bias. In addition, the stents originally implanted at the ISR target lesions may be first- or second-generation DES, which may affect plaque formation to some extent. Thus, the findings of this article cannot be generalized. Second, on the basis of the suggestion of Chinese experts, we set 30 mg/dL as the critical Lp (a) value in this study. However, this cut-off is not universal, and different results may be produced in regions and countries other than China. Third, we were unable to distinguish whether prior pharmacological therapy had an effect on the incidence and structural characteristics of ISNA and TCFA in patients with ISR. In particular, the inconsistent use of statins and antiplatelet agents may influence the differences in TCFA and other vulnerable plaque characteristics in targeted vessels between the two patient groups. Fourth, OCT has intrinsic limitations in the qualitative analysis of restenotic tissues, as recently reported by Lutter et al. Its image features are not completely consistent with pathological results [27], thus creating a risk of misjudgment of pathological features. As a result, further exploration combined with near-infrared spectroscopy or other technical means is warranted.

## Conclusion

In patients with ISR, the incidence of OCT-ISNA and OCT-TCFA was significantly associated with their Lp (a) level. Therefore, a high Lp (a) level is an important factor in the increased vulnerability of ISNA plaques.

**Supplementary Fig. 1. Prevalence of ISNA (A) and TCFA (B)**.

According to Lp (a) quartile level, ISR lesion were divided into four groups: (a) Lp (a) ≤ 8.48 mg/dL; (b) 8.48 mg/dL < Lp (a) ≤ 23.23 mg/dL; (c) 23.23 mg/dL < Lp (a) ≤ 46.25 mg/dL; (d) Lp (a) > 46.25 mg/dL. OCT-ISNA, in-stent neoatherosclerosis on OCT images; OCT-TCFA, thin-cap fibroatheroma on OCT images.

## Electronic supplementary material

Below is the link to the electronic supplementary material.

Supplementary Material 1
