# Supplementary material for: Lipoprotein (a) is related to In-Stent neoatherosclerosis incidence rate and plaque vulnerability: Optical Coherence Tomography Study
Source: Int J Cardiovasc Imaging. 2022 Oct 31;39(2):275–84. doi: 10.1007/s10554-022-02736-3 (PMC9870965; doi:10.1007/s10554-022-02736-3)
Supplement: Supplementary file 1 — Supplementary Material 1 [file 10554_2022_2736_MOESM1_ESM.docx]

Supplementary Material

1. **Supplementary Table 1. OCT analysis of culprit lesions according to Lp (a) quartile**

|  | Lp (a) ≤ 8.48  (n=31) | 8.48 < Lp (a) ≤ 23.23  (n=32) | 23.23 < Lp (a) ≤ 46.25  (n=31) | Lp (a) > 46.25  (n=31) | *P* value |
| --- | --- | --- | --- | --- | --- |
| Distal reference lumen area, mm^2^ | 4.9 (3.9, 6.4) | 5.0 (4.0, 5.9) | 4.9 (3.7, 6.4) | 6.0 (4.4, 7.2) | 0.319 |
| Proximal reference lumen area, mm^2^ | 6.1 (4.4, 7.8) | 6.5 (5.9, 7.9) | 7.1 (5.6, 9.1) | 6.8 (6.2, 9.1) | 0.315 |
| Minimum lumen area, mm^2^ | 2.2 (1.4, 2.9) | 2.1 (1.3, 3.1) | 2.0 (1.3, 2.5) | 1.9 (1.4, 3.0) | 0.867 |
| Minimum lumen diameter, mm^2^ | 1.7 (1.4, 1.9) | 1.6 (1.3, 2.0) | 1.6 (1.3, 1.8) | 1.5 (1.3, 1.9) | 0.864 |
| Minimum stent area, mm^2^ | 6.2 (4.7, 6.8) | 6.3 (4.8, 7.4) | 6.8 (5.9, 8.1) | 6.9 (6.0, 8.8) | 0.123 |
| Maximal NIH, % | 0.6 (0.5, 0.8) | 0.6 (0.4, 0.8) | 0.7 (0.6, 0.8) | 0.7 (0.5, 0.8) | 0.594 |
| Mean intimal thickness | 0.5 (0.4, 0.7) | 0.6 (0.4, 0.8) | 0.7 (0.5, 0.8) | 0.7 (0.5, 0.9) | 0.369 |
| Neoatherosclerosis | 13 (41.9) | 17 (53.1) | 27 (87.1) | 29 (93.5) | <0.001 |
| Lipid plaque | 9 (29.0) | 16 (50.0) | 26 (83.9) | 29 (93.5) | <0.001 |
| Maximum lipidic arc, ° ±SD | 240.0 (135.5, 330.0) | 178.8 (120.5, 267.6) | 271.5 (203.0, 360.0) | 305.0 (210.6, 360.0) | 0.017 |
| TCFA | 0 (0) | 3 (9.4) | 12 (38.7) | 9 (29.0) | <0.001 |
| Plaque rupture | 2 (6.5) | 3 (9.4) | 3 (9.7) | 7 (22.6) | 0.245 |
| Calcific plaque | 4 (12.9) | 3 (9.4) | 4 (12.9) | 2 (6.5) | 0.797 |
| Fibrous plaque | 18 (58.1) | 15 (46.9) | 4 (12.9) | 2 (6.5) | <0.001 |
| Thrombus | 5 (16.1) | 5 (15.6) | 4 (12.9) | 6 (19.4) | 0.922 |
| Neointimal macrophages | 0 (0) | 1 (3.1) | 10 (32.3) | 10 (32.3) | <0.001 |
| Microvessels | 5 (16.1) | 1 (3.1) | 14 (45.2) | 10 (32.3) | 0.001 |

Values are n (%) or median with first and third quartiles. NIH: neointimal hyperplasia; TCFA: thin-cap fibro-atheroma.

1. **Supplementary Figure 1. Prevalence of ISNA (A) and TCFA (B)**


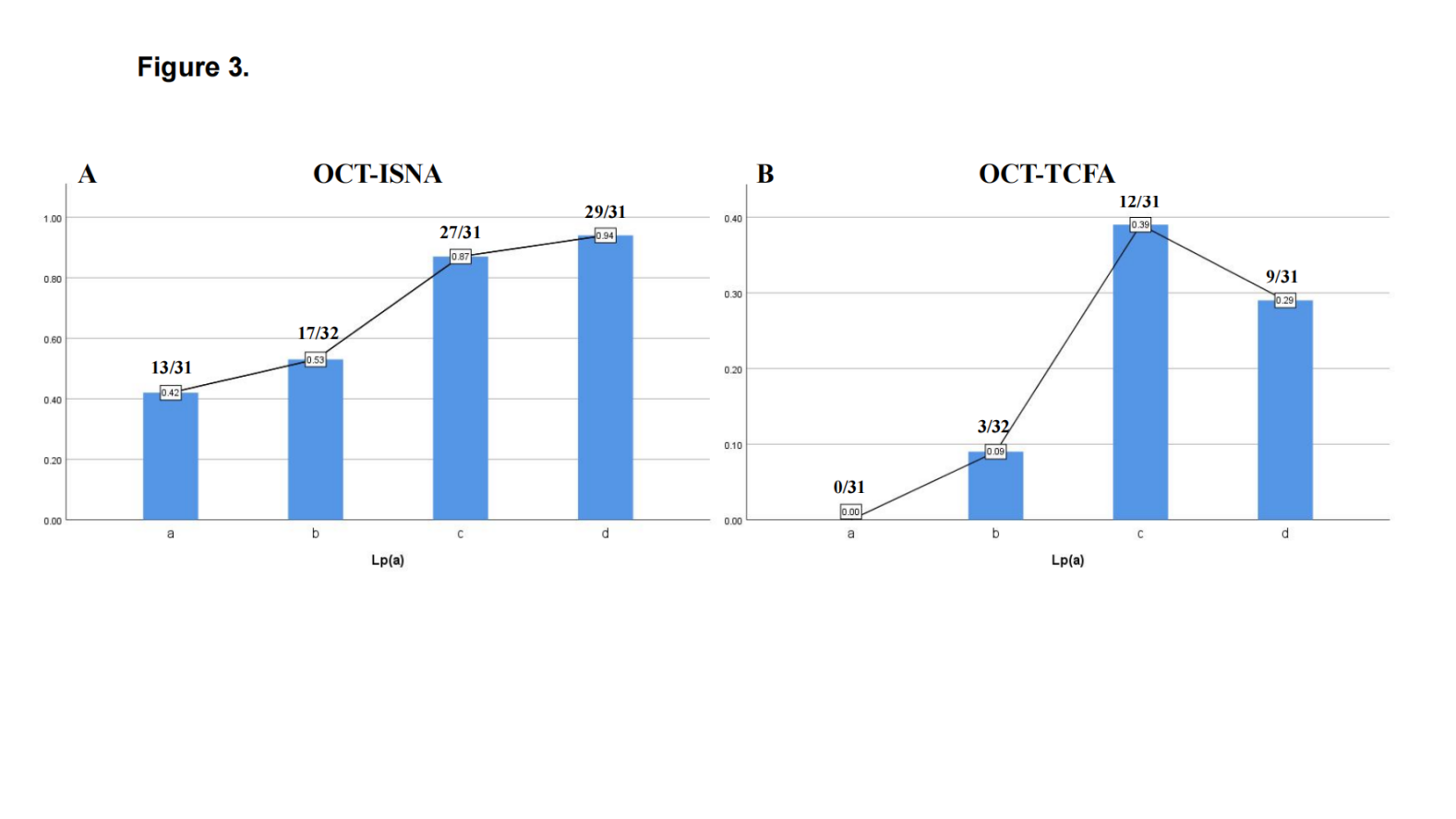
According to the Lp (a) quartile level, ISR lesions were divided into four groups: a) Lp (a) ≤ 8.48, b) 8.48 < Lp (a) ≤ 23.23, c) 23.23 < Lp (a) ≤ 46.25, and d) Lp (a) > 46.25. ISNA, in-stent neoatherosclerosis; TCFA, thin-cap fibroatheroma.
